# Phenotypic plasticity vs. local adaptation in quantitative traits differences of *Stipa grandis* in semi-arid steppe, China

**DOI:** 10.1038/s41598-018-21557-w

**Published:** 2018-02-16

**Authors:** Shao-bo Gao, Li-dong Mo, Li-hong Zhang, Jian-li Zhang, Jian-bo Wu, Jin-long Wang, Nian-xi Zhao, Yu-bao Gao

**Affiliations:** 1 Department of Plant Biology and Ecology, College of Life Science, Nankai University, Tianjin, 300071 P.R. China; 2 College of Agronomy & Resources and Environment, Tianjin Agricultural University, Tianjin, 300384 P.R. China

## Abstract

Whether plants are able to adapt to environmental changes depends on their genetic characteristics and phenotypic plastic responses. We investigated the phenotypic responses of 7 populations of an important dominant species in semi-arid steppe of China - *Stipa grandis*, and then distinguished which adaptive mechanism(s), phenotypic plasticity or local adaptation, was/were involved in this species to adapt to environmental changes. (1) All traits were significantly influenced by the interaction of population and growth condition and by population in each condition, and inter-population variability (CV_inter_) was larger in the field than in the common garden for 8/9 traits, indicating that both phenotypic plasticity and genetic differentiation controlled the phenotypic differences of *S. grandis*. (2) From a functional standpoint, the significant relationships between the values of traits in the common garden and the environmental variables in their original habitats couldn’t support local habitat adaptation of these traits. (3) Low CV_intra_, low quantitative differentiation among populations (*Q*_*ST*_), and low plasticity shown in the western populations indicated the very low adaptive potential of *S. grandis* to environmental changes. (4) From the original habitats to the common garden which is far away from *S. grandis* distribution region, positive phenotypic responses were found in several populations, indicating that some original habitats have become unfavorable for *S. grandis*.

## Introduction

Environmental changes, such as climatic changes or anthropogenic activities, would be expected to shift plants’ distributions as species expand in newly favorable areas or decline in increasingly unfavorable/hostile locations^1^. Whether a plant in terrestrial ecosystems is able to adapt to the environmental changes depends on its population genetic characteristics and phenotypic plastic responses^2,3^. The importance of genetic characteristics to predict distribution shifts is advancing^4^. However, the potential for phenotypic plastic responses has often been neglected even though understanding evolutionary potential of a species is limited without considering its phenotypic responses.

A deep understanding of phenotypic plastic responses of a species is necessary to forecast its full potential to adapt and/or evolve to changing conditions^5^. Likewise, because of the increasing impact of environmental changes on plants’ distribution shifts, there has been increasing interest in identifying which adaptive mechanism(s) of phenotypic responses, phenotypic plasticity, or local adaptation (adaptive genetic changes), or combination of these two mechanisms, help(s) them to adapt to environmental changes^6,7^. Phenotypic plasticity can be distinguished from genetic differentiation which includes local adaptation (adaptive genetic changes) and maladaptive or non-adaptive genetic changes, depending on whether quantitative traits differences among populations *in situ* disappear by raising individual plants from these populations under the same conditions (i.e. in common gardens)^8–10^. Moreover, if quantitative traits differences *in situ* show larger than those in common gardens, it might be controlled by the combination of phenotypic plasticity and genetic differentiation^11^. Furthermore, ecologically meaningful correlations between the values of quantitative traits in the common garden and the environmental variables in their original habitats could predict local adaptation (adaptive genetic changes) to the selection of environmental changes^11–13^. What’s more, how a trait varies within and among populations is critical to determine the potential of a species/population to perform along environmental gradients^8^.

Due to climate changes and anthropogenic activities, steppes are becoming fragmented and degraded, especially in arid and semi-arid areas^14^. Comparing with tree species^2,15–18^, only a few studies paid attentions to phenotypic plastic responses of steppe species and local adaptation is less common^10,19,20^. Given steppe species could not adapt to the rapid environmental changes, their distributions will be greatly influenced. Therefore, more studies are needed to know about the phenotypic plastic responses of steppes species to environmental changes in order to protect the structures and functions of grassland communities.

*Stipa grandis* steppe is the most common, representative and stable community of typical steppe in Euro-Asian Steppe^21^. However, the distribution region of *S. grandis* has rapidly changed due to fragmentation and degradation by climate changes and anthropogenic activities in the past decades, showing a pattern of eastward migration^22^. Mode of reproduction can influence distribution shifts by affecting evolutionary potential and dispersal capacity^3^. *S. grandis* is self-compatible^23^, therefore, the fragmentation and degradation of habitats would enhance its inbreeding and enlarge population genetic drift, decrease population genetic diversity, then affect its evolutionary potential to environmental changes^24^. In our pervious study, amplified fragment length polymorphism (AFLP) markers were used to analyze its population genetic characteristics based on 7 populations across its distribution region in semi-arid steppe of China^25^. In this study, exactly the same 7 populations (Table 1) were chosen to analyze its phenotypic plastic responses because the combination of genetic and phenotypic analysis could help us to forecast a species’ full potential to adapt to rapid environmental changes^26^. Nine quantitative traits of individual plants in these populations were measured in a field (*in situ*) and in a common garden, and the environmental variables including geographic and bioclimatic variables in their original habitats were collected to use for trait – environment correlation analysis. In order to test the evolutionary potential and distinguish phenotypic adaptive mechanism(s) of *S. grandis* to the environmental changes across its main distribution region, we tested reaction norms of these quantitative traits from their original habitats to the common garden, estimated traits differences among populations in each condition, calculated intra (inter) - population variability (CV_intra_ and CV_inter_) for every trait, related the values of quantitative traits with the environmental variables in their original habitats in each condition, and calculated quantitative differentiation among populations (*Q*_*ST*_) for every trait examined in the common garden.

**Table 1:** Geographical coordinate, 19 bioclimatic variables and the two first principal component scores for these bioclimatic variables of sampling sites.

Variable		Bayantuohai	Holingole	Bayanwula	Wuliyasitai	East-Xilinhot	West-Xilinhot	Bieligutai
**Geographical coordinate**								
Longitude (°E)		119.55	119.72	117.73	117.03	116.61	115.58	115.07
Latitude (°N)		49.07	45.43	44.63	45.57	44.24	43.89	44
Altitude (m)		951	950	1104	998	1121	1073	1149
**Bioclimatic variable**								
Annual Mean Temperature		−1.2	0.7	0.7	0.6	0.4	1.6	0.8
Mean Diurnal Range (Mean of monthly (max - min)), BIO2		12.7	13.5	13.3	13.7	13.6	14	14
Isothermality (BIO2/BIO7) (*100)^⁑^		2.2	2.6	2.6	2.5	2.6	2.6	2.5
Temperature Seasonality (standard deviation *100)^⁑^		158.1	136.3	135.5	149.7	138.3	143.3	144.3
Max Temperature of Warmest Month, BIO5		25.7	25	24.9	26.9	25.2	27	26.4
Min Temperature of Coldest Month, BIO6^⁑^		−30.9	−25.7	−25.4	−27.5	−26.4	−26.6	−27.6
Temperature Annual Range (BIO5-BIO6), BIO7		56.6	50.7	50.3	54.4	51.6	53.6	54
Mean Temperature of Wettest Quarter		17.9	17.5	17.2	18.9	17.3	18.9	18.3
Mean Temperature of Driest Quarter		−19.4	−17.6	−17.7	−19.7	−18.4	−18.2	−19.1
Mean Temperature of Warmest Quarter		17.9	17.5	17.2	18.9	17.3	18.9	18.3
Mean Temperature of Coldest Quarter		−22.8	−17.6	−17.7	−19.7	−18.4	−18.2	−19.1
Annual Precipitation^※^		358	416	352	263	325	269	253
Precipitation of Wettest Month^※^		103	131	104	75	93	75	70
Precipitation of Driest Month^※^		3	3	3	2	3	2	2
Precipitation Seasonality (Coefficient of Variation)		111	115	109	112	106	107	105
Precipitation of Wettest Quarter^※^		249	297	243	183	221	183	174
Precipitation of Driest Quarter		12	10	9	6	9	6	6
Precipitation of Warmest Quarter^※^		249	297	243	183	221	183	174
Precipitation of Coldest Quarter		12	10	9	6	9	6	6
PC scores for 19 bioclimatic variables	PC-1 (56.36% variance)	0.66	1.27	0.79	−1.04	0.39	−1.06	−1
	PC-2 (36.12% variance)	−2.09	0.66	0.77	−0.37	0.44	0.52	0.07

Variables whose absolute values of factor loading are above 0.90 are marked by ^※^for PC-1 and ^⁑^for PC-2, respectively.

## Results

### Principal component analysis (PCA) for bioclimatic variables

The first 2 principal components summarized 89.86% of the overall variation among the 19 layers. PC-1 and PC-2 explained 53.56% and 36.12% variance, respectively (Table 1). PC-1 could be thought of as precipitation component because the variables with high loadings (> 0.9) on PC-1 were annual precipitation, precipitation of wettest and driest month, precipitation of wettest and warmest quarter, and PC-2 could be thought of temperature component because the variables with high loadings (> 0.9) on PC-2 were isothermality, temperature seasonality, and mean temperature of coldest quarter (Table 1).

### Phenotypic plasticity and reaction norms

**Figure 1:**
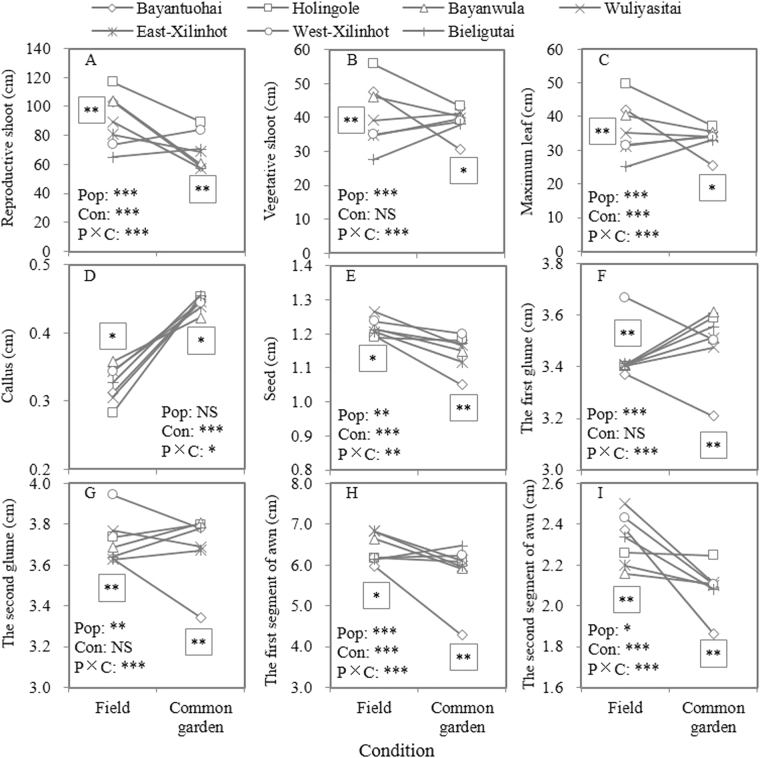
Results of analysis of variance on quantitative traits of different *S. grandis* populations both in the field and in the common garden. *P < 0.05, **P < 0.01, ***P < 0.001. Stars without border indicate the significance of factors’ effects by two-way analysis of variance, while stars with square border indicate the significance of difference among populations in the field (left) or in the common garden (right) by one-way analysis of variance.

The phenotypic plastic responses of different *S. grandis* populations were expressed by their slopes from original habitat to the common garden and their plasticity was shown by the absolute values of the slopes. The interaction of population (P) and growth condition (C) showed significant effects (*P* < 0.05) on all 9 traits, that is to say, there were significant different reaction norms among populations (Figs. 1A–I), indicating that different *S. grandis* populations showed different phenotypic plasticity to adapt to the changing conditions and that there was a genetic basis for their phenotypic plasticity. Both negative and positive responses were found for 6 traits (Figs. 1A–C, F–H), only negative responses for length of seed and the second segment of awn (Figs. 1E, I), and only positive responses for length of callus (Fig. 1D). Bayantuohai population (the most eastern population in this study) showed the highest absolute values of slopes for all traits, with 8 negative and 1 positive value (Fig. 1D). Bieligutai population showed positive slopes for 7 traits (Figs. 1A–D, F–H) and negative slopes for 2 traits (Figs. 1E, I). The middle and western populations, such as Bieligutai, West-Xilinhot, East-Xilinhot showed relatively lower absolute values of slopes than the eastern populations.

### Phenotypic differences in the field

In the field, all quantitative traits showed significant differences (*P* < 0.05) among populations (left in Figs. 1A–I).

**Figure 2:**
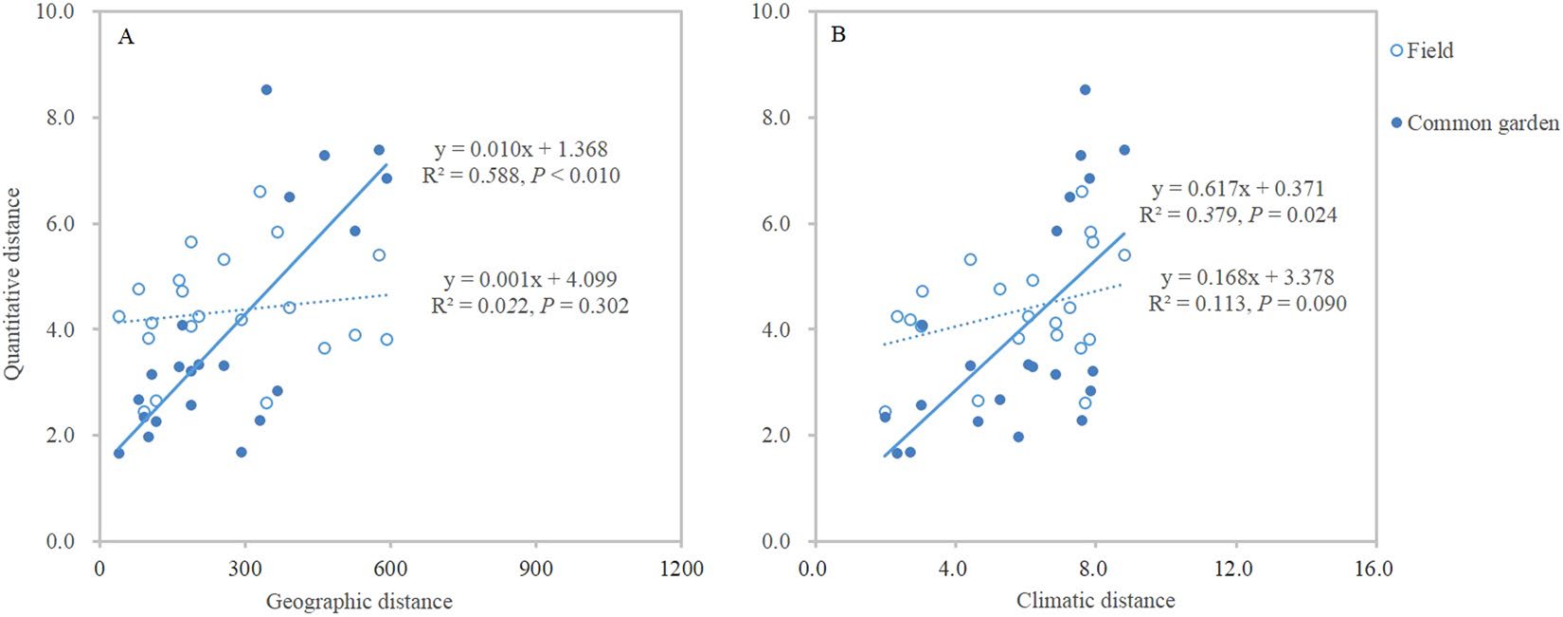
Relationships between quantitative distances and geographic distances (km) (**A**) climatic distances (**B**) of pair-wise *S. grandis* populations both in the field and in the common garden.

Three growth related traits showed significantly (*P* < 0.01) positive relationships with longitude, height of reproductive shoot showed a significantly negative relationship with PC-1 score (precipitation component) (*P* < 0.05), and height of vegetative shoot and length of the maximum leaf showed significantly negative relationships with altitude (*P* < 0.05). No significant relationships were found between seed related traits and any environmental variable (*P* > 0.05) (Table 2). In addition, non-significant relationships were found between field-quantitative and geographic distances (R^2^ = 0.022, *P* = 0.302) (Fig. 2A), between field-quantitative and climatic distances (R^2^ = 0.113, *P* = 0.090) by Mantel’s tests (Fig. 2B).

**Table 2:** Spearman’s correlations between values of quantitative traits of different *S. grandis* populations and environmental variables in their original habitats.

Traits	Field					Common garden				
	Longitude	Latitude	Altitude	PC scores for 19 bioclimatic variables		Longitude	Latitude	Altitude	PC scores for 19 bioclimatic variables	
				PC-1	PC-2				PC-1	PC-2
Height of reproductive shoot	0.964**	0.679	−0.714	0.821*	0.357	−0.025	−0.412	−0.064	0.094	0.536
Height of vegetative shoot	0.964**	0.714	−0.893**	0.679	0.179	−0.146	−0.701	0.148	0.006	0.858*
Length of the maximum leaf	0.964**	0.714	−0.893**	0.679	0.179	−0.202	−0.785*	0.279	0.049	0.945**
Length of callus	−0.491	−0.6	0.709	−0.218	0.436	0.105	0.356	−0.47	−0.253	−0.49
Length of seed	−0.505	−0.27	0.306	−0.739	−0.018	−0.493	−0.797*	0.276	−0.442	0.782*
Length of the first glume	−0.73	−0.674	0.674	−0.468	0.524	−0.345	−0.856*	0.489	−0.019	0.956**
Length of the second glume	−0.09	−0.252	−0.288	−0.414	0.342	−0.452	−0.894**	0.484	−0.19	0.944**
Length of the first segment of awn	−0.162	−0.126	0.234	−0.252	0.252	−0.66	−0.936**	0.563	−0.451	0.847*
Length of the second segment of awn	−0.214	0.143	−0.321	−0.714	−0.643	−0.117	−0.704	0.122	0.057	0.878**

*,** indicate significant correlations at the 0.05, 0.01 level, respectively.

CV_intra_ ranged from 0.065 to 0.192 and CV_inter_ ranged from 0.070 to 0.264. CV_intra_ was a little lower than CV_inter_ for all traits, with significant differences (*P* < 0.001) for 3 growth related traits and non-significant differences for 6 seed related traits (Table 3).

**Table 3:** Intra -population variability (CV_intra_) and inter-population (CV_inter_) of 9 quantitative traits of different *S. grandis* populations measured both in the field and in the common garden and quantitative differentiation among populations (*Q*_*ST*_) of these 9 traits measured in the common garden.

Traits	Field				Common garden				
	CV_intra_		CV_inter_	P value	CV_intra_		CV_inter_	P value	Q_ST_
Height of reproductive shoot	0.112	<	0.217	0.000	0.166	<	0.227	0.01	0.274
Height of vegetative shoot	0.15	<	0.264	0.000	0.149		0.174	0.155	0.188
Length of the maximum leaf	0.153	<	0.262	0.001	0.156	<	0.182	0.042	0.199
Length of callus	0.192		0.205	0.526	0.061		0.077	0.16	0.061
Length of seed	0.065		0.07	0.195	0.053	<	0.063	0.047	0.096
Length of the first glume	0.094		0.098	0.447	0.075		0.089	0.169	0.113
Length of the second glume	0.094		0.1	0.425	0.077		0.09	0.146	0.048
Length of the first segment of awn	0.139		0.15	0.494	0.109	<	0.131	0.05	0.033
Length of the second segment of awn	0.127		0.14	0.29	0.105		0.124	0.214	0.037

### Phenotypic differences in the common garden

All quantitative traits examined in the common garden showed significant differences (*P* < 0.05) among populations (right in Figs. 1A–I). *Q*_*ST*_ of these traits ranged from 0.033 to 0.274 (Table 3).

Five traits (lengths of the maximum leaf, seed, the first and second glume, the first segment of awn) showed significantly (*P* < 0.05) negative relationships with latitude. Seven traits, excluding height of reproductive shoot and length of callus, showed significantly (*P* < 0.05) positive relationships with PC-2 scores (temperature component) (Table 2). Mantel’s tests showed significant relationships between common garden - quantitative and geographic distances (R^2^ = 0.588, *P* = 0.010) (Fig. 2A), and between common garden - quantitative and climatic distances (R^2^ = 0.379, *P* = 0.024) (Fig. 2B).

CV_intra_ ranged from 0.053 to 0.166 and CV_inter_ ranged from 0.063 to 0.227. CV_intra_ was a little lower than CV_inter_ for all traits, with significant differences (*P* < 0.05) for height of reproductive shoot, length of the maximum leaf, seed, and the first segment of awn (Table 3). CV_inter_ in the common garden was lower than in the field for all traits except the height of reproductive shoot (Table 3).

## Discussion

From original habitats to common garden, reaction norms of *S. grandis*  were significantly (*P* < 0.05) different for all 9 traits as shown by the significance of the interaction of population and growth condition, traits differences among populations were significant (*P* < 0.05) in each condition (Fig. 1), and CV_inter_ in the field was larger than in the common garden for 8/9 traits (Table 3). These results indicated that both phenotypic plasticity and genetic differentiation controlled the phenotypic differences of different *S. grandis* populations and suggested the genetic basis of phenotypic plasticity of *S. grandis*^11,27^. But, we did not provide determination proofs for local adaptation (adaptive genetic changes) of *S. grandis* populations although some significant trait – environment relationships were found. For example, regarding  9 quantitative traits measured in the common garden, five traits showed significantly negative relationships with latitude and 7 traits showed significantly positive relationships with PC-2 score (temperature component) by Spearman’s correlation analysis (Table 2). Moreover, significant relationships were found between common garden-quantitative and geographic (climatic) distances by Mantel’s tests (Fig. 2). From a functional standpoint, smaller sizes may be favored in drier habitats, as growth related traits tested in the field were shown (Fig. 1; Table 2), because smaller leaves provide less surface area for transpiration water loss and smaller organ and plant size can reduce developmental time^26,28^. However, the significant relationships mentioned above suggested that the organs or plant sizes of *S. grandis* increased with the increase of the temperature. That is to say, *S. grandis* had larger organs or plant sizes in relatively drier habitats (Fig. 1; Tables 1 and 2). Therefore, these significant trait-environment relationships did not show ecologically meaningful trends to support that local adaptation (adaptive genetic changes) helped *S. grandis* populations to adapt to their local conditions.

Maladaptive or non-adaptive genetic changes could occur as a result of genetic drift or founder effect, or as a result of stress, nutrient limitation^7^. Both fragmental habitats and distribution shifts could contribute to non-adaptive genetic changes by increasing population genetic drift or environmental stress. In recent decades, because of less raining and intense human activities, *S. grandis* communities were fragmented and degraded, and as a result, they were replaced by other communities (e.g. *S. krylovii* community) and the distribution region of *S. grandis* has eastward shifted^22^. In the present study, the common garden site was chosen as an unfavorable or a hostile condition because it is beyond the distribution region of *S. grandis*^22^. From their original habitats to the common garden, the eastern populations, such as Bayantuhai, showed negative phenotypic plastic responses for most traits (negative slopes in Fig. 1), demonstrating that the common garden condition was not as favor as their original habitats; however, populations from the western region, such as Bieligutai and West-Xilinhot, showed positive phenotypic plastic responses for most traits (positive slopes in Fig. 1), indicating that their original habitats were more unfavorable (hostile) than the common garden condition. These results provided some proofs for the possibility of maladaptive or non-adaptive genetic changes affecting *S. grandis*’ phenotypic difference among populations as well for the eastward shift of *S. grandis* distribution region^7,22^. In addition, according to the theory of Merilä and Crnokrak^29^, *Q*_*ST*_ = *F*_*ST*_, *Q*_*ST*_ > *F*_*ST*_ or *Q*_*ST*_ < *F*_*ST*_ is predicted if trait differentiation is under neutral, or under directional selection for different local optima (like another expression of adaptive genetic changes), or under homogenizing selection, respectively. In this study, the result that *Q*_*ST*_ of 8 traits was lower than *F*_*ST*_ value (0.2431) by AFLP markers^25^ indicated that homogenizing selection rather than directional selection played an important role in affecting the quantitative trait differentiation among *S. grandis* populations.

Variability within and between populations could also help plant to track environmental changes. In the present study, several CV_intra_ showed significantly lower than CV_inter_ (Table 3), and both CV_intra_ and CV_inter_ were relatively lower than other species reported^8,10^. Furthermore, plasticity of the western populations of *S. grandis* was lower than that of the eastern populations. The most eastern population -Bayantuohai had the highest absolute values of slopes for all traits (Fig. 1). Besides, compared with other outcrossing or perennial grasses, *S. grandis* had a relatively low population genetic diversity^25^. Relatively low CV_intra_ of quantitative traits, low plasticity of some populations and low population genetic diversity would seriously hamper the adaptive capacity of *S. grandis* to environmental changes, such as climate changes and intense anthropogenic activities.

Summarily, phenotypic plasticity rather than local adaptation (adaptive genetic changes) played an important role in helping *S. grandis* populations to adapt to environmental changes. Bearing in mind non-adaptive genetic changes and low adaptive capacity of *S. grandis* populations, some measures should be carried out to protect their habitats in order to decrease environmental stress or unfavorable/hostile environmental conditions, and then gradually decrease population genetic drift and enhance population genetic diversity, finally improve population evolutionary potential to environmental changes and maintain ecological functions of the communities.

## Materials and Methods

### Species and sampling sites

*S. grandis* is the most important dominant and constructive species of the climax community in semi-arid steppe of China, therefore, its distribution shifts and population changes have great effects on community structure and function. We have studied its genetic characteristics based on 7 *S. grandis* populations which covers its main distribution region (115–120°E, 43–50°N), and in this study, we selected exactly the same 7 populations to analyze their phenotypic plastic responses from their original habitats to a common garden. A detailed description of sampling sites could be found in Wu *et al*.’s literature^25^.

### Common garden experiment

Common garden experiment was carried out in an open experiment field at Nankai University in Tianjin which is far away from *S. grandis* distribution region and was thought as a hostile growth condition for *S. grandis*. Soil was collected from semi-arid steppe of China and filled in 0–30 cm in the experiment field, soil C, N, P contents showed similar values with the mean of their original habitats^10^, but average annual precipitation (550–680 mm) and annual temperature (12.3 °C) were higher than their original habitats (Table 1). Seeds (actually caryopses) of *S. grandis* sorted by maternal plant were used for germination in January 4, 2008. One progeny per maternal plant was randomly chosen and transplanted at the center of an open spacing of 30 cm × 30 cm in April, 2009. The experiment was designed as a completely randomized block design, with 3 individuals per population in each block and 105 individuals in five blocks totally. During the experiment, all individuals grew under natural conditions except that weeding was carried out once a week.

### Quantitative trait measurements

Three growth related traits, height of reproductive shoot and vegetative shoot, and length of the maximum leaf, were measured during the flowering period, and 6 seed related traits, length of callus, seed, the first and the second glume, the first and the second segment of awn were measured at the end of the growing season. The measurements were performed for 50 *S. grandis* individuals per population in the field in 2007 and 15 individuals per population in the common garden in 2011, respectively. Besides, values of 6 seed related traits were the mean from 10 spikelets within individual.

### Bioclimatic variables collection

Nineteen bioclimatic variables representative of the original habitats’ climatic conditions from 1950 to 2000 (Table 1) were analyzed for this study. These bioclimatic variables could be obtained from the WorldClim database freely by geographical coordinate^30^, and detailed descriptions and calculations about them could be found in James *et al*.’s literature^31^.

### Statistical analysis

Quantitative data meet assumption of normality and homogeneity of variance, therefore, they do not have to be transformed before data analysis. First, two-way analysis of variance (IBM, Armonk, NY) was conducted to investigate the effect of block on values of quantitative traits examined in the common garden, with block and population as fixed factors. Results showed that values were not influenced by block and population × block interaction. Therefore, we did not have to think about the block factor when we analyzed data examined in the common garden. Second, in order to examine the differences of phenotypic plasticity and reaction norms among populations, two-way analysis of variance (IBM, Armonk, NY) was conducted to investigate effects of population, growth condition and their interaction on quantitative data, with population and growth condition as fixed factors. Third, based on significant interactions of population and growth condition, we further analyzed trait differences among populations in each condition (field or common garden) by one-way analysis of variance (IBM, Armonk, NY), and got within-population variance (σ_*w*_) and between-population variance (σ_*P*_), then calculated quantitative differentiation among populations (*Q*_*ST*_) by formula *Q*_*ST*_ = σ_*P*_^2^/(σ_*P*_^2^ + 2σ_*w*_^2^)^29,32^. Fourth, intra-population variability (CV_intra_) and inter-populations variability (CV_inter_) were calculated as the ratio of SD_within population_ to population mean and the ratio of SD_between population_ to overall mean, respectively. “SD” is the abbreviation of “standard deviation”. Significant difference between CV_intra_ and CV_inter_ was tested by one sample *t*-test, with CV_inter_ as test value (IBM, Armonk, NY).

In order to reduce dimensionality from initial 19 bioclimatic variables by geographical coordinate, principal component analysis (PCA) was used, and variable factor loadings, cumulative proportions of the total variance and scores of the first 2 principal components for each population were calculated (IBM, Armonk, NY). Furthermore, relationships between values of quantitative traits and the environmental variables in their original habitats were analysed by Spearman’s correlation analyses (IBM, Armonk, NY). It should be noted that environmental variables included geographic data (longitude, latitude, altitude) and climatic data (the first two principal components scores for 19 bioclimatic variables) in this study.

Population pair-wise distance matrix based on 19 bioclimatic variables or quantitative data collected in each condition were calculated by Euclidean’s distance coefficient after standardization of data, respectively (IBM, Armonk, NY). Population pair-wise geographic distance matric were estimated in Google Earth. Relationships between quantitative and geographic distances, and between quantitative and climatic distances were examined by Mantel’s tests (3000 permutations) in NTSYS-pc software^33^.
